# Prevalence of Type 2 Diabetes among Patients Visiting Nepal Police Hospital in Kathmandu: A Descriptive Cross-sectional Study

**DOI:** 10.31729/jnma.5734

**Published:** 2021-01-31

**Authors:** Bibek Rajbhandari, Sundar Prasad Hyoju, Lisasha Poudel, Anurag Adhikari, Badri Rijal, Pramod Joshi

**Affiliations:** 1 Nepal Police Hospital, Kathmandu, Nepal; 2 Dhulikhel Hospital-Kathmandu University Hospital, Nepal; 3 Independent Data Analyst and Researcher; 4 Department of Orthopedics, All Nepal Hospital Private Limited, Samakushi, Kathmandu, Nepal; 5 Department of Orthopedics, Seti Provincial Hospital, Dhangadhi, Nepal

**Keywords:** *diabetes mellitus*, *hospital*, *Nepal*, *prevalence*

## Abstract

**Introduction:**

The growing prevalence of diabetes mellitus (DM) is a critical threat for global health, including in Nepal, especially in Kathmandu District, where diabetic patients are increasing in hospitals catastrophically. This study tends to assess the prevalence rate of type 2 diabetes among admitted patients visiting a hospital in Kathmandu.

**Methods:**

An electronic chart review was done to assess the prevalence of type 2 diabetes in Nepal Police hospital, Kathmandu, Nepal. Ethical approval was taken from the Nepal Health Research Council. Considering eligibility criteria, 8631 cases from 4 May 2018 to 31 August 2020 were observed. The calculated sample size was 500. However, all diabetic cases, i.e., 576, were processed and analyzed using Python and later visualized using MS Excel.

**Results:**

The overall prevalence rate of type 2 diabetes was 6.67%. The prevalence rate was seen higher among males (7.5%). Similarly, a high prevalence rate was seen among 64-73 years, i.e., 15.10%.

**Conclusions:**

The findings showed a high prevalence rate of type 2 diabetes. Older age groups are at high risk. Urgent public health interventions including lifestyle modification measures are required to reduce the extra burden of type 2 diabetes.

## INTRODUCTION

The burden of diabetes continues to pose a significant problem in both low and high-income countries. In 2019, the global prevalence of diabetes was projected at 9.3 percent (463 million people), increasing to 10.2 percent (578 million) by 2030 and 10.9 percent (700 million) by 2045.^1^ It is estimated that the biggest rise would take place in regions where economies are transiting from low-to middle-income status. Of the two types of diabetes, type 2 accounts for nearly 90% - 95% of all diagnosed diabetes cases.^2^

Nepal is undergoing an epidemiological transition phase experiencing a double burden, from a higher prevalence of communicable diseases to non-communicable diseases.^3^ Type 2 Diabetes is one of the prevalent NCDs seen in Nepal. Diabetes cases have been rapidly registered in hospitals, with 199113 cases as of FY 2017/18.^4^ This study aims to find out the prevalence of Type 2 Diabetes among patients visiting a hospital in Kathmandu, Nepal.

## METHODS

A study was conducted in Nepal Police Hospital to assess the prevalence rate of type 2 diabetes. An electronic chart review was done of 576 diabetic patients admitted to the hospital from 4 May, 2018 to 31 August, 2020. An approval letter was taken from the hospital. Ethical approval was obtained from Nepal Health Research Council (Reg no.2082) before data collection.

Sample Size calculation:

Sample size was calculate using below formula

n = sample size
p = (50%) 0.5
q = 1-p
Z = 1.96 for Confidence Interval of 95%
e = margin of error, 5%

$$\begin{array}{ll} \text{n} & ={\text{Z}}^{\text{2}}\text{p(}1-\text{p)}/{\text{e}}^{\text{2}}\\ & ={\left(1.96\right)}^{2}\times (0.5)\times (1-0.5)/{\left(0.05\right)}^{\text{2}}\\ & =384.16\\ & =\text{385 (Approx.)} \end{array}$$

Taking high non-response rate, i.e., 30%, the total sample size was 500.

Inclusion criteria: Patients aged 14-93 years; admitted cases from 4 May, 2018 to 31 August, 2020.

Exclusion criteria: ICU admitted cases, pediatric population, missing values of data; redundant data, diabetic associated with other endocrinal pathology were excluded.

**Data Pre-Processing:**

The initial cluttered data was cleaned with the process of data mining techniques as follows:

1. Removal of unwanted observations, which included, falls under exclusion criteria.
2. Handling the inconsistent data by converting them into a common format or consistent format by using python script. The ages that had other characters rather than numbers were removed using regular expressions from the python script.

**Data Analysis and Visualization:**

1. The cleaned data were then grouped under the features of age and gender.
2. The ratio of the overall Diabetes Mellitus (Type 2 Diabetes) observations to the overall observations of the clinical records was calculated. The prevalence of the overall observations was the percentage of the ratio calculated.
3. The same analysis was done based on age groups and genders whose outcomes were individual prevalence of the age groups and the genders.
4. The above was visualized in a bar graph and a line graph using MS Excel.

## RESULTS

The total prevalence rate of type 2 diabetes in Nepal Police Hospital was 576 (6.67%).

**Table 1:** Overall Prevalence rate:

Total Observations	Total DM Observations	Prevalence Rate (%)
8631	576	6.67

A high prevalence rate was seen among male (7.4%) as compared with the female (5.5%)

**Table 2:** Prevalence rate based on gender:

Gender	Total Observations	DM Observations	Prevalence Rate (%)
Female	3765	211	5.6
Male	4867	365	7.5

**Figure 1:**
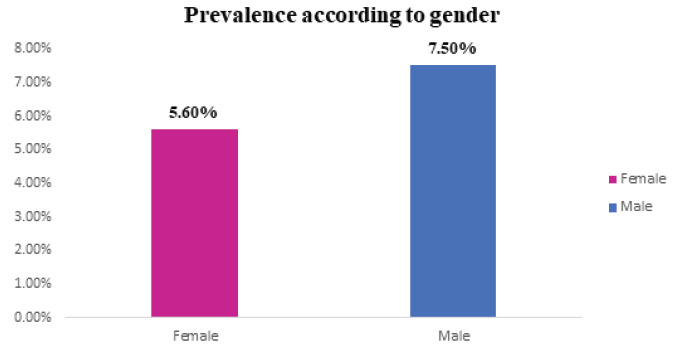
Prevalence Rate based on gender

The highest prevalence rate was seen among 64-73 years, i.e., 15.10%.

**Table 3:** Prevalence rate based on age.

Age Group	Total Age Group Observations	DM Age Group Observations	Prevalence Rate (%)
14-23	864	11	1.27
24-33	2522	57	2.26
34-43	1752	75	4.28
44-53	1069	112	10.48
54-63	959	113	11.78
64-73	927	140	15.10
74-83	428	58	13.55
84-93	110	10	9.09

**Figure 2:**
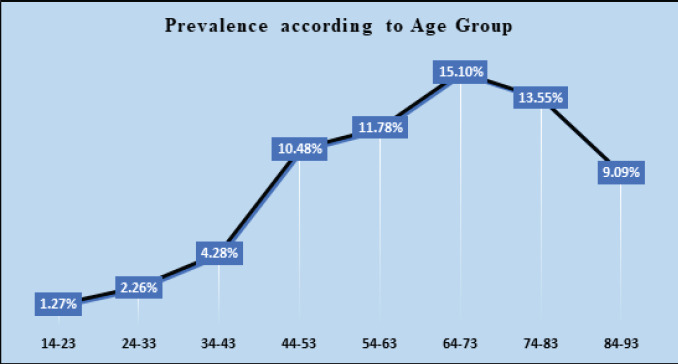
Prevalence Rate based on age group.

Using an electronic chart review, the current study presented the prevalence of Type 2 diabetes among patients admitted to the hospital. The result showed a 6.67 percent of prevalence rate. This was similar to a IDF report that reported the national prevalence rate of 4% among 20-70 years in Nepal.^1^

The finding of this study were consistent to studies conducted by Baral (5.29%),^5^ Dhimal (8.5%),^6^ Ono K (9.5%).^7^ In contrast the prevalence was lower than previous studies from Nepal; Mehta (10.9%)^8^, Bishal (11.7%),^9^ Shrestha (19%),^10^ Singh (14.7%),^11^ Dhungana(10.5%).^12^

Likewise, the prevalence for diabetes was higher than in the earlier nationwide STEPS survey, which was 3.6%.^13^ The pooled prevalence from a systematic review study of Nepal showed a higher prevalence of diabetes (8.5%) but low awareness (52.7%) among diabetes patients.^14^

A meta-analysis done by Gyawali B et al. 2015, showed the prevalence of type 2 diabetes in Nepal that ranged from a minimum of 1.4 percent to a high of 19.0 percent, including the combined prevalence of type 2 diabetes, which was 8.4 percent. The study also presented high prevalence rate among female which contrast with our study.^3^

The prevalence of type 2 diabetes (6.67%) in this study was comparatively lower than the national prevalence obtained in many other South Asian countries, including Bangladesh 8.1%, Bhutan 8.7%, India 8.9%, Maldives 7.3%, Mauritius 25.3%, and Sri Lanka 8.7%.^1^

Age is one of the most prominent risk factors for type 2 diabetes, and the disease prevalence in older groups is quite high.^15^ There is the highest risk of developing type 2 diabetes in middle-aged and older adults. A study reported the age-wise prevalence of diabetes in the Asian population. Among Chinese and Japanese subjects, the prevalence of diabetes grew with age and peaked at 70-89 years of age, but among Indian subjects peaked at 60-69 years of age, followed by a decline at 70 years of age.^16^ This is similar to our study that showed a peak point at 64-73 years of age.

It has been found that cases of diabetes are increasingly registered in hospitals of Nepal. In 2010, diabetes accounted for almost 10 percent of admissions to the medical ward at TU Teaching Hospital, Kathmandu, which is higher than our study.^17^

## CONCLUSIONS

The study revealed a high prevalence rate of type 2 diabetes among admitted patients of Nepal Police hospital. The result also showed a higher prevalence among the older age group and male gender. Since if diagnosed and treated early, diabetes mellitus is a preventable condition. Therefore, routine screening shall be implemented to reduce the national burden and further improve the quality of life.
